# Clinical Implication of DNA Damage Response Genes in Advanced Gastric Cancer Stage IV and Recurrent Gastric Cancer Patients After Gastrectomy Treated Palliative Chemotherapy

**DOI:** 10.7150/jca.81632

**Published:** 2023-05-05

**Authors:** Jeong Eun Kim, Song Ee Park, Hee Jun Kim, In Gyu Hwang

**Affiliations:** 1 Department of Internal Medicine, Chung-Ang University College of Medicine, Seoul, Korea; 2 Chung-Ang University Integrated Oncology and Palliative Care Research Institute, Seoul, Korea

**Keywords:** Gastric cancer, dMMR, Immunohistochemistry, MLH1, MSH2

## Abstract

**Purpose:** This study aimed to investigate the relationship between DNA damage response (DDR)-related protein expression and the clinical outcomes of patients with gastric cancer stage IV and recurrent advanced gastric cancer patients after gastrectomy treated with palliative first-line chemotherapy.

**Materials and Methods:** A total of 611 gastric cancer patients underwent D2 radical gastrectomy at Chung-Ang University Hospital between January 2005 and December 2017, of which 72 patients who received gastrectomy treatment with palliative chemotherapy were enrolled in this study. We performed the immunohistochemical assessment of MutL Homolog 1 (MLH1), MutS Homolog 2 (MSH2), at-rich interaction domain 1 (ARID1A), poly adenosine diphosphate-ribose polymerase 1 (PARP-1), breast cancer susceptibility gene 1 (BRCA1), and ataxia-telangiectasia mutated (ATM) using formalin-fixed paraffin-embedded samples. In addition, Kaplan-Meier survival analysis and Cox regression models were used to evaluate independent predictors of overall survival (OS) and progression-free survival (PFS).

**Results:** Among the 72 patients studied, immunohistochemical staining analysis indicated deficient DNA mismatch repair (dMMR) in 19.4% of patients (n = 14). The most common DDR gene with suppressed expression was PARP-1 (n = 41, 56.9%), followed by ATM (n = 26, 36.1%), ARID1A (n = 10, 13.9%), MLH1 (n = 12, 16.7%), BRCA1 (n = 11, 15.3%), and MSH2 (n = 3, 4.2%). HER2 (n = 6, 8.3%) and PD-L1 (n = 3, 4.2%) were expressed in 72 patients. The dMMR group exhibited a significantly longer median OS than the MMR proficient (pMMR) group (19.9 months vs. 11.0 months; hazard ratio [HR] 0.474, 95% confidence interval [CI] = 0.239-0.937, P = 0.032). The dMMR group exhibited a significantly longer median PFS than the pMMR group (7.0 months vs. 5.1 months; HR= 0.498, 95% CI = 0.267-0.928, P = 0.028).

**Conclusions:** Of stage IV gastric cancer and recurrent gastric cancer patients who underwent gastrectomy, the dMMR group had a better survival rate than the pMMR group. Although dMMR is a predictive factor for immunotherapy in advanced gastric cancer, further studies are needed to determine whether it is a prognostic factor for gastric cancer patients treated with palliative cytotoxic chemotherapy.

## Introduction

Gastric cancer is the fifth most common cancer with the fourth most cancer-related mortality rate worldwide 1. In patients with human epidermal growth factor receptor 2 (HER2) negative stage 4 gastric cancer, the standard first-line treatment is fluoropyrimidine plus platinum-based chemotherapy 2. These patients have a poor prognosis with a survival period of less than one year. The recent CHECKMATE 642 study reported that a programmed death (PD)-1 inhibitor, nivolumab, combined with chemotherapy provided superior overall survival (OS) than chemotherapy alone in previously untreated advanced gastric cancer patients 3. However, despite its potential relevance, no clinically relevant survival prognostic marker for gastric cancer has been identified yet.

The DNA damage response (DDR) is activated within a cell cycle checkpoint when DNA damage occurs 4. Cancer cells with a deficiency in DDR have continuous growth and survival. In a previous study, targeting the DDR pathway of gastric cancer had survival benefit 5. DDR-related proteins, such as ataxia-telangiectasia mutated protein (ATM), breast cancer susceptibility gene (BRCA1) 6, poly [ADP-ribose] polymerase 1 (PARP-1) 7, AT-rich interaction domain 1A (ARID1A) 8, and MutL Homolog 1 (MLH)1 and MSH2 9 facilitate cancer cell survival and proliferation and the evasion of physiological cell cycle checkpoints.

PARP1 is a polymerase that functions in the DNA repair response to DNA damage by conjugated ADP from NAD+ to target proteins such as p53 and histones 10. Especially, when there are defects in DNA repair caused by mutations of BRCA1/2, the inhibition of PARP1 results in unrepairable DNA and the apoptosis of cancer cells 11. ATM is a phosphatidylinositol 3-kinase-like kinase family member. Together with another kinase, ataxia telangiectasia mutated (ATM), it acts as a central regulator of the cellular response to DNA damage. ARID1A regulates cellular responses through interactions with ATM 12.

DDR expression is involved in the progression of cancers and resistance to anti-cancer drugs 13. It was suggested that DDR expression could be a therapeutic target for the treatment of malignant tumors. In the phase 3 GOLD study, the amount of ATM was confirmed by an ATM immunohistochemical (IHC) assay using formalin-fixed, paraffin-embedded gastric carcinoma tissues. However, this trial did not show a survival advantage in gastric cancer after first-line chemotherapy with olaparib 14.

DDR expression was correlated with an improved response to cisplatin-based chemotherapy in other cancers 15. Genomic alterations in the DNA response and repair-associated genes predicted responses and clinical benefits after cisplatin-based chemotherapy. MLH1, MSH2, PMS2, and MSH6 are defined as mismatch repair (MMR) expression proteins. MSI-H/dMMR gastric cancers were associated with a better OS compared with proficient MMR (pMMR) in a recent meta-analysis 16, 17.

In this study, we investigated the relationship between the expression of DDR and survival to determine the survival-associated prognostic potential of DDR-related proteins in stage IV gastric cancer patients and recurrent advanced gastric cancer patients after gastrectomy treated with palliative first-line chemotherapy.

## Materials and Methods

### Patients

A total of 611 gastric cancer patients underwent D2 radical gastrectomy at Chung-Ang University Hospital between January 2005 and December 2017, of which 72 who received gastrectomy and were treated with palliative chemotherapy were enrolled. The inclusion criteria were as follows: a pathologically confirmed gastric adenocarcinoma, recurrent or metastatic gastric cancer; treated at least one cycle of first-line palliative chemotherapy. Exclusion criteria were immunohistochemical staining cannot be performed. The clinical data of patients were collected from their medical records, including sex, age, chemotherapy regimen, and survival. The cancer staging was performed according to the 7th edition of the American Joint Committee on Cancer. This study was approved by the Institutional Review Board of Chung-Ang University Hospital (IRB number: 1981-005-382).

### Immunohistochemistry

We performed the immunohistochemical assessment of MLH1, MSH2, ARID1A, PARP-1, BRCA1, and ATM using formalin-fixed paraffin-embedded samples. The mismatch repair proteins MLH1 and MSH2 were scored based on the following threshold: positive when staining was detected in 10% or more of tumor cell nuclei; negative when staining was detected in less than 10% of tumor cell nuclei.

PARP-1 staining was scored based on the staining intensity as follows: 0 (negative), 1 (weak), 2 (moderate), and 3 (strong). The percentage of staining distribution of each marker within the tumor cells was recorded. A histochemical (H) score was then calculated as follows: (1 percentage weak), (2 percentage moderate), and (3 percentage strong). The H-score is representative of the overall staining intensity with a range from 0 to 300. PARP-1 staining was scored as follows: positive or high expression, staining achieving H-scores of more than 175; negative or low expression, staining achieving H-scores of less than 175. Receiver operating characteristic (ROC) curve analysis in other study included gastric cancer was performed to determine an optimal cutoff H score of 175 for PARP-1 expression.^18^.

ARID1A staining was scored as follows: negative, undetectable; positive, no loss and focal loss. BRCA1 staining was scored as follows: negative, staining in less than 5% of tumor cell nuclei; positive, staining in more than 5% of tumor cell nuclei. An ATM assay was evaluated based on the nuclear signal, with the percentage of weakly stained cells over a range of 0-300. A dichotomous classification system was devised whereby the cases were classified as follows: negative, intensity staining in ≤ 10% of cancer cells (H-score ≤ 10); or positive, staining in more than 10% of cancer cells.

HER2 IHC staining was scored as follows: 0 and 1+ were considered negative; 2+ was considered unable to confirm HER2 overexpression; and 3+ indicated HER2 overexpression.

### Statistical analyses

All statistical analyses were performed using the Statistical Package for Social Sciences (SPSS) version 26.0 (IBM Corp., Armonk, NY, USA). We analyzed the patients' clinicopathological features and prognoses by SPSS. In addition, Kaplan-Meier survival analysis and Cox regression models were used to evaluate independent predictors of OS and progression-free survival (PFS). Hazard ratios (HRs) and their corresponding 95% confidence intervals (CI) were stratified using a Cox proportional hazards regression model. P-values <0.05 were considered statistically significant. GraphPad Prism 9.0 was used to generate the survival curves.

## Results

### Patients characteristics

The baseline characteristics of the patients are shown in Table 1. The median age was 64 years (range: 36-88 years) and there were 49 (68.1%) males and 23 (31.9%) females. According to pT stage, two patients (2.8%) were in T1 stage. Three patients (4.2%) were in T2, 26 patients (36.1%) were in T3, and 41 patients (56.9%) were in T4. Lymph node metastasis was observed in 68 patients (94.4%). With respect to TNM stage, 28 patients (38.9%) patients were in *de novo* stage IV and 44 patients (66.1%) had recurrent cancer after gastrectomy. All patients were histologically confirmed to have adenocarcinoma. Adjuvant chemotherapy was given to 36 individuals who underwent gastrectomy (50.0%).

### Expression of DDR-related proteins

**Figure 1:**
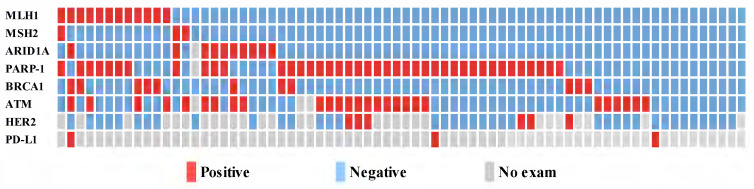
Relationship between the expression level of the DDR bio markers, HER2 and PD-L1 (n=72).

Among the 72 patients studied, immunohistochemical staining demonstrated deficient DNA mismatch repair (dMMR) in 19.4% (n = 14) of patients. The most common DDR gene with suppressed expression was PARP-1 (n = 41, 56.9%), followed by ATM (n = 26, 36.1%), ARID1A (n = 10, 13.9%), MLH1 (n = 12, 16.7%), BRCA1 (n = 11, 15.3%), and MSH2 (n = 3, 4.2%) (Fig. 1).

### Correlation between DDR-related protein expression and survival

**Figure 2:**
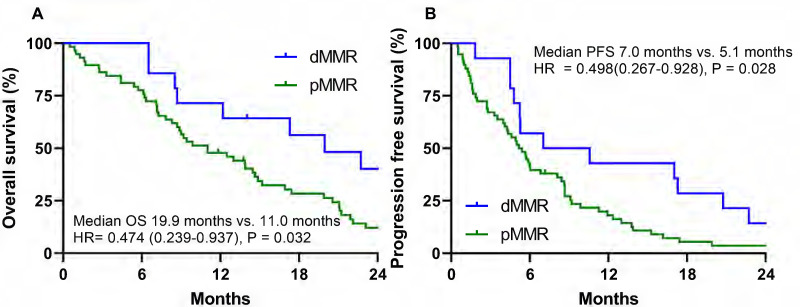
The dMMR group in stage IV gastric cancer patients is associated with a significantly better overall survival (OS) **(A)** and progression-free survival (PFS) **(B)**.

The cutoff time for the analyses was January 2020, resulting in a median follow-up of 82.4 months (95% CI = 31.2-133.4 months) including the death of 64 patients (88.9%). The median OS and PFS were 12.5 months (95% CI = 7.9-17.0 months) and 5.4 months (95% CI = 4.3-6.4 months), respectively. During the follow-up period, 70 patients (97.2%) relapsed or died. The dMMR group exhibited a significantly longer median OS than the MMR proficient (pMMR) group (19.9 months vs. 11.0 months; HR 0.474, 95% CI = 0.260-0.937, P = 0.032). The dMMR group exhibited a significantly longer median PFS than the pMMR group (7.0 months vs. 5.1 months; HR= 0.498, 95% CI = 0.267-0.928, P = 0.028) (Fig. 2).

The univariate OS analysis of the potential prognostic impact of the clinicopathological parameters identified dMMR (HR = 0.474, P = 0.032) as a significant predictor for OS (Table 2). In the multivariate OS analysis, dMMR (HR = 0.395, P = 0.029) was the only significant prognostic factor. There was no significant correlation between OS and age, sex, ARID1A, PARP-1, BRCA1, ATM, adjuvant chemotherapy, and palliative chemotherapy regimen (FOLFOX (fluorouracil, leucovorin, and oxaliplatin)/CAPOX (capecitabine and oxaliplatin) vs. S-1 (oral prodrug of 5-fluorouracil) vs. other regimens).

Similar to the analysis of OS, dMMR was revealed to be a possible prognostic factor of the clinicopathological parameters by the univariate PFS analysis (HR = 0.498, P = 0.028) (Table 3). In the multivariate PFS analysis, dMMR (HR= 0.365, P = 0.012) was the only significant prognostic factor. There was no significant correlation between PFS and age, sex, ARID1A, PARP-1, BRCA1, ATM, adjuvant chemotherapy, and palliative chemotherapy regimen.

### Impact of dMMR expression on the palliative cytotoxic chemotherapy regimen

Palliative first-line chemotherapy was given to 72 patients. Of these, 38 (52.8%) received palliative oxaliplatin-based chemotherapy, 11 (15.3%) received S-1 monotherapy, and 23 patients (31.9%) received another chemotherapy regimen involving S-1 combined with oxaliplatin, ramucirumab combined with paclitaxel, and capecitabine alone. There was no significantly different median OS correlation between palliative chemotherapy regimens (FOFOX/CAPOX vs. S-1 vs. other regimens: 13.9 months vs. 11.0 months vs. 9.0 months, P = 0.719). There was no significantly different median PFS correlation between palliative chemotherapy regimens (FOFOX/CAPOX vs. S-1 vs. other regimens: 5.8 months vs. 5.6 months vs. 4.3 months, P = 0.506).

**Figure 3:**
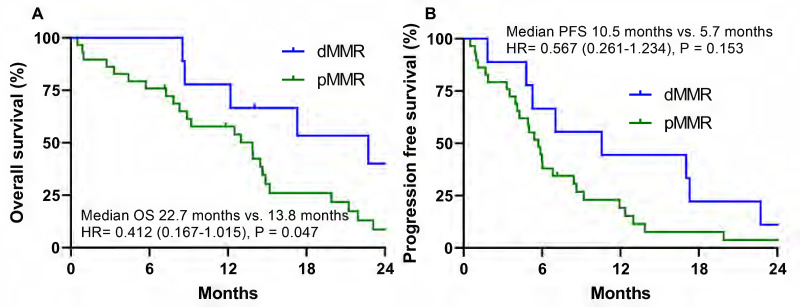
Overall survival (A) and progression free survival (B) according to dMMR vs. pMMR in the oxaliplatin-based palliative first-line chemotherapy group with stage IV gastric cancer.

In the oxaliplatin-based palliative first-line chemotherapy group, the dMMR group had a significantly different median OS than the pMMR group (22.7 months vs. 13.8 months, HR =0.412 (0.167-1.015), P = 0.047) (Fig. 3A). In the oxaliplatin-based palliative first-line chemotherapy group, the dMMR group had a numerically longer PFS than the pMMR group (10.5 months vs. 5.7 months, HR =0.567 (0.261-1.234), P = 0.153), which did not reach statistical significance (Fig. 3B). In contrast to the oxaliplatin group, the S-1 group in the dMMR group had a numerically shorter OS (6.5 months vs. 11.0 months, HR = 1.484 (0.358-6.143), P = 0.586) and PFS compared to the pMMR group, which did not reach statistical significance (4.5 months vs. 5.6 months, HR = 2.213 (0.457-10.708), P = 0.324).

We conducted additional an analysis in the dMMR group in order to identify the predictors of immune checkpoint inhibitors. However, we are unable to draw any conclusions because the patient numbers were too low.

## Discussion

This study presented the results of the immunohistochemical assessment of the expression of DDR protein in 72 gastric cancer patients with stage IV and recurrent gastric cancer after gastrectomy. We analyzed the DDR gene expression in surgical samples from patients who underwent gastric cancer surgery, and confirmed a relationship with the expression of each gene examined. We also analyzed the relationship between survival and chemotherapy regimen. The results showed that the dMMR group was associated with a good prognosis.

According to statistics from The Cancer Genome Atlas project, the rate of MSI-H stomach cancer is 22%, which is comparable to the dMMR gene expression (19.4%) in our study 19. MSI-H gastric cancer patients had a good prognosis. Other studies reported approximately 5% of patients had dMMR gene expression 20. However, in another study, patients who had metastasis at the time of surgery were excluded and they reported the dMMR gastric cancer stage IV rate was low, and for those with stage I to III, patients had a dMMR gene expression of 22%-43%. In our study, of the MMR protein expressions we wanted to measure (MLH1, MSH2, MSH6, and PMS2), MSH6 and PMS2 could not be tested. Thus, the estimated dMMR expression in this study might be lower than expected. However, a study of other cancer types such as colon cancer, only measured MLH1 and MSH2 21. MSH6 and PMS2 were present at a low frequency, suggesting there was no significant difference in dMMR protein expression.

Our research indicated that patients with dMMR gene expression had a superior OS and PFS to patients with pMMR gene expression. In the era of immunotherapy using cancer drugs, the dMMR group is known to respond well to immune checkpoint inhibitors. In the KEYNOTE-061 study, the 12-month OS rate for pembrolizumab in MSI-H tumors was 73% (95% CI = 44%-89%) compared with 25% (95% CI = 6%-50%) for chemotherapy alone. As with the 12-month OS rate, the median PFS in MSI-H tumors was 17.8 months (95% CI = 2.7 months to not reached) for the pembrolizumab group vs. 6.6 months (95% CI = 4.4-8.3 months) for the cytotoxic chemotherapy group 22, 23. In the CHECKMATE-649 study, the objective response rate of nivolumab combined with chemotherapy was higher (58%) in comfort patients with a combined positive score (CPS) > 5 or higher than 48% in the chemotherapy only group 3.

Cohen et al. emphasized the importance of platinum-based adjuvant chemotherapy in colon cancer stage 3 patients with MSI-H, as the oxaliplatin combined fluoropyrimidine chemotherapy regimen significantly improved the OS after performing adjuvant chemotherapy 15. A meta-analysis of gastric cancer showed that adjuvant chemotherapy performed in patients with gastric cancer with dMMR/MSI-H significantly improved their DFS and OS 17. Based on this, our study also confirmed that dMMR patients had a significantly improved OS and PFS compared to the pMMR group, regardless of the palliative first-line cytotoxic chemotherapy regimen.

This study reports a significant improvement in the OS and PFS in the oxaliplatin-based chemotherapy group compared to the dMMR and pMMR groups. However, there was no difference in the OS and PFS according to the chemotherapy regimen in the multivariate analysis. This suggests that gastric cancer patients with dMMR have a better prognosis than patients with pMMR, regardless of the chemotherapy regimen.

## Conclusions

Of stage IV gastric cancer and recurrent gastric cancer patients who underwent gastrectomy, the dMMR group had a better survival rate than the pMMR group. Although dMMR is a predictive factor for immunotherapy in advanced gastric cancer patients, further studies are needed to determine whether it is a prognostic factor for gastric cancer patients treated with palliative cytotoxic chemotherapy.

## Figures and Tables

**Table 1:** Baseline characteristics.

Characteristics	Total (n = 72)
Age (years)	
Median	64
Range	36-88
Age ≥ 65 years	33 (45.8%)
Sex, n (%)	
Male	49 (68.1%)
Female	23 (31.9%)
Invasion depth	
T1	2 (2.8%)
T2	3 (4.2%)
T3	26 (36.1%)
T4	41 (56.9%)
Lymph node metastasis	
Negative	4 (5.6%)
Positive	68 (94.4%)
Histological subtype	
Well- differentiated adenocarcinoma	1 (1.3%)
Moderately differentiated adenocarcinoma	26 (36.1%)
Poorly differentiated adenocarcinoma	40 (55.6%)
Signet-ring cell carcinoma	5 (6.9%)
Surgery	
Total gastrectomy	36 (50.0%)
Subtotal gastrectomy	36 (50.0%)
Adjuvant chemotherapy	
No	31 (43.1%)
Yes	41 (56.9%)
Stage at diagnosis	
*De novo* stage IV	28 (38.9%)
Recurrent cancer after gastrectomy	44 (61.1%)
Palliative 1st chemotherapy regimen	
CAPOX or FOFLOX	38 (52.8%)
S-1	11 (15.3%)
Other	23 (31.9%)

TNM, tumor-node-metastasis; CAPOX, capecitabine and oxaliplatin; FOLFOX, 5-fluorouracil, leucovorin, and oxaliplatin; S-1, oral prodrug of 5-fluorouracil.

**Table 2:** Univariate and multivariate Cox regression models used to analyze factors affecting overall survival.

		Univariate Cox regression model		Multivariate Cox regression model	
		HR (95% CI)	P-value	HR (95% CI)	P-value
Age (years)	< 65 vs. ≥ 65	1.433 (0.871-2.358)	0.156	1.746 (0.969-3.146)	0.064
Sex	Male vs. female	0.799 (0.464-1.374)	0.417	0.559 (0.296-1.054)	0.072
MMR	pMMR vs dMMR	0.474 (0.239-0.937)	0.032	0.395 (0.171-0.911)	0.029
ARID1A	High vs. low	1.032 (0.635-1.677)	0.899	1.002 (0.417-2.407)	0.997
PARP-1	High vs. low	1.006 (0.604-1.675)	0.981	1.023 (0.527-1.987)	0.945
BRCA1	High vs. low	1.190 (0.603-2.349)	0.377	0.711 (0.319-1.585)	0.405
ATM	High vs. low	0.838 (0.490-1.433)	0.518	1.085 (0.579-2.033)	0.798
Previous adjuvant chemotherapy	No vs. yes	0.991 (0.599-1.640)	0.971	0.773 (0.420-1.423)	0.408
Chemotherapy regimen	FOLFOX/CAPOX	Reference		Reference	
	S-1	0.977 (0.478-1.994)	0.948	0.772 (0.319-1.868)	0.566
	Other regimen	1.235 (0.711-2.145)	0.454	0.771 (0.398-1.493)	0.440

dMMR, deficient DNA mismatch repair; pMMR, DNA mismatch repair proficient; ARID1A, AT-rich interaction domain 1; PARP-1, Poly adenosine diphosphate-ribose polymerase 1; BRCA1, Breast cancer susceptibility gene 1; ATM, ataxia-telangiectasia mutated; CAPOX, capecitabine and oxaliplatin; FOLFOX, 5-fluorouracil, leucovorin, and oxaliplatin; S-1, oral prodrug of 5-fluorouracil.

**Table 3:** Univariate and Multivariate Cox regression models used to analyze factors affecting progression survival.

		Univariate Cox regression model		Multivariate Cox regression model	
		HR (95% CI)	P-value	HR (95% CI)	P-value
Age (years)	< 65 vs. ≥ 65	0.959 (0.596-1.543)	0.863	0.790 (0.427-1.460)	0.451
Sex	Male vs. female	0.799 (0.476-1.340)	0.395	0.595 (0.317-1.117)	0.106
MMR	pMMR vs dMMR	0.498 (0.267-0.928)	0.028	0.365 (0.166-0.804)	0.012
ARID1A	High vs. low	1.037 (0.527-2.043)	0.916	0.907 (0.400-2.005)	0.815
PARP-1	High vs. low	0.928 (0.574-1.500)	0.759	1.168 (0.641-2.128)	0.612
BRCA1	High vs. low	0.876 (0.458-1.676)	0.689	1.102 (0.507-2.395)	0.806
ATM	High vs. low	1.158 (0.702-1.910)	0.565	0.787 (0.442-1.400)	0.415
Previous adjuvant Chemotherapy	No vs. yes	0.986 (0.610-1.593)	0.953	0.595 (0.317-1.117)	0.106
Chemotherapy regimen	FOLFOX/CAPOX	Reference			
	S-1	0.893 (0.442-1.805)	0.753	1.000 (0.391-2.556)	1.000
	Other regimen	1.301 (0.769-2.203)	0.327	1.160 (0.599-2.245)	0.661

dMMR, deficient DNA mismatch repair; pMMR, DNA mismatch repair proficient; ARID1A, AT-rich interaction domain 1; PARP-1, Poly adenosine diphosphate-ribose polymerase 1; BRCA1, Breast cancer susceptibility gene 1; ATM, ataxia-telangiectasia mutated; CAPOX, capecitabine and oxaliplatin; FOLFOX, 5-fluorouracil, leucovorin, and oxaliplatin; S-1, oral prodrug of 5-fluorouracil.

## Abbreviations

ARID1A: AT-rich interaction domain 1
ATM: ataxia-telangiectasia mutated
BRCA1: Breast cancer susceptibility gene 1
CAPOX: capecitabine and oxaliplatin
DDR: DNA damage response
FOLFOX: 5-fluorouracil, leucovorin, and oxaliplatin
HER2: human epidermal growth factor receptor 2
HRs: Hazard ratios
dMMR: deficient DNA mismatch repair
pMMR: DNA mismatch repair proficient
OS: overall survival
PARP-1: Poly adenosine diphosphate-ribose polymerase 1
PFS: progression-free survival
S-1: oral prodrug of 5-fluorouracil
TNM: tumor-node-metastasis
